# Dietary Quantity and Diversity among Anemic Pregnant Women in Madura Island, Indonesia

**DOI:** 10.1155/2019/2647230

**Published:** 2019-09-30

**Authors:** Rian Diana, Ali Khomsan, Faisal Anwar, Dyan Fajar Christianti, Rendra Kusuma, Riris Diana Rachmayanti

**Affiliations:** ^1^Department of Nutrition, Faculty of Public Health, Universitas Airlangga, Campus C Mulyorejo, Surabaya, East Java 60115, Indonesia; ^2^Department of Community Nutrition, Faculty of Human Ecology, IPB University, Bogor, Indonesia; ^3^Graduate School (Nutrition), IPB University, Bogor, Indonesia; ^4^Department of Physical and Sport Education, STKIP PGRI Sumenep, Sumenep, Indonesia; ^5^Department of Health Promotion and Behavioral Science, Faculty of Public Health, Universitas Airlangga, Surabaya, Indonesia

## Abstract

Dietary diversity and quantity are important for pregnant women, particularly anemic pregnant women. This study aimed to analyze the association between dietary quantity and diversity among anemic pregnant women. This cross-sectional study was conducted in 2017 at Madura Island, Indonesia, and involved 152 anemic pregnant women. Hemoglobin concentration was analyzed by the cyanmethemoglobin method. Dietary quantity was measured by the 2 × 24 h recall. Dietary diversity was determined by Minimum Dietary Diversity for Women of Reproductive Age (MDD-W). Spearman's rank association was performed to analyze the association between dietary diversity and quantity. The median of hemoglobin concentration was 10.1 g/dL, and 57.2% pregnant women had mild anemia. Most of the pregnant women had low adequacy levels of energy and macro- and micronutrients (except for iron). More than half (57.9%) of anemic pregnant women had reached minimum dietary diversity. Family size (*p*=0.048) and gestational age (*p*=0.004) had negative associations with dietary diversity. Dietary diversity had positive associations with energy (*p*=0.029), protein (*p*=0.003), vitamin A (*p*=0.001), vitamin C (*p*=0.004), and zinc (*p*=0.015) adequacy levels. Dietary diversity had no significant association with calcium (*p*=0.078) and iron adequacy level (*p*=0.206). High prevalence of mild and moderate anemia was found among pregnant women in their third trimester. Anemic pregnant women already consumed food with minimum dietary diversity but did not meet dietary quantity. Increasing dietary quantity is a priority for anemic pregnant women.

## 1. Introduction

Maternal anemia is a major public health problem in the world, particularly in developing countries [1]. Globally, anemia prevalence among pregnant women was declining in the last 25 years (1990–2016) from 43.4% to 40.1% [2]. In contrast to this worldwide trend, there was a significant increase in the prevalence of anemia among pregnant women in Indonesia in the last five years, from 37.1% in 2013 to 48.9% in 2018 [3]. This condition may result in adverse maternal and fetal consequences. Anemia, directly and indirectly, attributes to maternal and perinatal death [1]. The risk of preterm birth was five times higher in anemic pregnant women than nonanemic women [4].

The high prevalence of anemia in pregnant women reflects a wide range of nutritional deficiencies. Socioeconomic status, gestational age, low dietary diversity, low dietary quantity, and intake of iron supplement during pregnancy are risk factors for anemia [5, 6]. Dietary diversification has been recommended for ensuring adequate nutrient intake of pregnant women, especially micronutrient adequacy [7].

Adequate nutrient intake and its association with nutritional status of pregnant women have been studied extensively [8–11]. Dietary diversity among pregnant women also has been studied in several countries [12, 13], including Indonesia [14]. On the other hand, no study was conducted on a specific high-risk population such as anemic pregnant women. Therefore, this study aimed to analyze the association between dietary diversity and dietary quantity among anemic pregnant women.

## 2. Methods

### 2.1. Study Design and Sampling

This study was conducted using a cross-sectional design in January-March 2017 at Sumenep District, Madura Island, Indonesia. The population were all anemic pregnant women in four local community health centers (252 anemic pregnant women), with 95% confidence level and margin of error 5%; therefore, 152 anemic pregnant women were recruited in this study. The sampling frame was taken from four local community health centers, and the sample was chosen by simple random sampling. Sample inclusion criteria were as follows: pregnant women aged 18–49 years, having anemia (Hb < 11 g/dL), not having a special diet, and willing to participate in this study by signing informed consent.

### 2.2. Data Collection

Hemoglobin concentration of the sample was assessed by researchers using the cyanmethemoglobin method. Blood sample was taken in the morning (07.00–10.00 AM) at village hall. Venous blood samples (5 ml) were collected by midwives, and professional health analysts measured hemoglobin concentration. One-on-one interview was done in the participants' house. Socioeconomic characteristics were collected using structured questionnaires. Dietary quantity was measured by the 2 × 24-h recall (nonconsecutive days) and then converted into nutrients by a trained nutritionist. The food picture book was used in food recall to help the participants calculate the food size. Meanwhile, dietary diversity was determined by Minimum Dietary Diversity for Women of Reproductive Age (MDD-W) based on 24-h dietary recall. MDD-W consisted of 10 food groups, namely, grains, white roots and tubers and plantains, pulses, nuts and seeds, dairy, meat, poultry, and fish, eggs, dark-green leafy vegetables, vitamin A-rich fruits and vegetables, and other vegetables and other fruits [7].

### 2.3. Data Analysis

The data were analyzed by using the IBM Statistical Package for Social Sciences (SPSS) program version 22. The descriptive statistics which included proportion, median, minimum, maximum, and interquartile range (IQR) were presented. The nutrient adequacy level was measured by comparing nutrient intake with Indonesian recommended dietary allowances (RDA) [15]. The Kolmogorov–Smirnov test showed that the data were not normally distributed (*p* < 0.05). Therefore, the association between socioeconomic characteristics, nutritional adequacy levels, and dietary diversity was analyzed by Spearman's rank correlation. The Mann–Whitney *U* test was used to analyze the difference of nutrient adequacy level and dietary diversity between mild and severe anemia.

The WHO [16] cutoff points for anemia were as follows: mild (10.0–10.9 g/dL), moderate (7.0–9.9 g/dL), and severe (<7.0 g/dL). Meanwhile, dietary diversity was categorized as low (<5 food groups) and high (≥5 food groups).

### 2.4. Ethical Approval

Ethical approval was obtained from the Health Research Ethics Committee, Faculty of Public Health, Universitas Airlangga No 1-KEPK. All participants had signed the inform consent before the data were collected.

## 3. Results

A total of 152 anemic pregnant women participated in this study. The median of hemoglobin concentration was 10.1 g/dL with minimum concentration 8.0 g/dL and maximum 10.9 g/dL. In terms of severity, more than half of pregnant women (57.2%) had mild anemia and the rest (42.8%) had moderate anemia. There were no severely anemic pregnant women in this study (Table 1). In general, the majority of pregnant women were 19–29 years, in their third trimester, having small family size and eating three times/day. Most of the pregnant women had basic and secondary education. Nonetheless, moderately anemic pregnant women had a higher proportion of maternal age less than 18 years old, basic education, meal frequency less than 2 times per day, and a lower proportion of tertiary education compared to mildly anemic pregnant women. The pregnancy stages show that, in trimester 1, pregnant women had a higher proportion of having mild anemia than older gestational age. In contrast, women in trimesters 2 and 3 had a higher proportion of suffering moderate anemia than in trimester 1. In total, moderately anemic pregnant women had a higher income than mildly anemic pregnant women (Table 1).

Pregnancy complication was not assed in this study. However, health complaints in pregnancy showed that moderately anemic mothers have a higher proportion of having health complaints than mildly anemic mothers (Table 2). Common complaints in pregnancy by participants were back pain, feeling tired, having upper respiratory tract infection, headache, nausea/vomiting, decreasing appetite, constipation, and swollen feet.

### 3.1. Dietary Quantity

Generally, all anemic pregnant women were unable to fulfill the Recommended Dietary Allowance (RDA) for energy, protein, and micronutrients from food consumption. Pregnant women with moderate anemia had a higher proportion of inadequate nutrient intake than mildly anemic pregnant women.  Table 3 shows that most of the pregnant women have low nutrition adequacy levels. The majority of anemic pregnant women had <70% energy adequacy level and <90% protein adequacy level. The protein adequacy level was slightly better compared to the energy adequacy level. Many of them had low micronutrient adequacy levels, such as vitamin A, vitamin C, calcium, and zinc. In contrast, most of them had a good iron adequacy level. The high iron adequacy level was mostly contributed from the consumption of iron-folic supplement; only 15–20% of RDA was fulfilled by the food. The Mann–Whitney test shows no significant differences of nutrient adequacy level among mild and moderate anemia pregnant women.

### 3.2. Dietary Diversity

In total, more than half of anemic pregnant women had a diverse diet (consumed ≥5 food groups). Sample with mild anemia (62.5%) had a better dietary diversity than moderate anemia (37.5%) (Table 4). Staple foods, particularly rice and corn, were consumed by all respondents. The consumption of animal source foods such as meat, poultry, fish, and other kinds of seafood was relatively high compared to egg, milk, and dairy product consumption. Animal source food that was often consumed was fish. Milk was the only animal source food rarely consumed by the pregnant women.

Pulses, particularly soybean in form of tempeh and tofu, were consumed more than nuts and seeds. Peanut in form of peanut sauce added in traditional mixed dishes was popular food among people in the study area. The low consumption of vegetables and fruits was found in this study. Only half of the anemic pregnant women consumed dark-green leafy vegetables. Meanwhile, other vegetables and fruits, including vitamin A-rich fruits and vegetables, were consumed by less than 30%. Dark-green leafy vegetables usually consumed by pregnant women were moringa leaves, spinach, water spinach, and cassava leaves. Other vegetables such as cucumber, bean sprouts, and cabbage were also popular among pregnant women. Vitamin A-rich fruits and vegetables most frequently consumed was the carrot (Table 4). The Mann–Whitney *U* test showed that pregnant women with mild anemia had better dietary diversity than moderate anemia (*p*=0.025). Mildly anemic pregnant women consume more eggs (60.3%), milk and dairy product (65%), dark-green leafy vegetables (62.8%), vitamin A-rich fruits and vegetables (70.3%), other vegetables (63.8%), and other fruits (61.7%) compared with moderately anemic pregnant women.

The Spearman rank test revealed that family size (*p*=0.004, *r* = −0.160) and gestational age (*p*=0.044, *r* = −0.164) were negatively associated with dietary diversity. Meanwhile, energy, protein, vitamin A, vitamin C, and zinc adequacy levels were positively associated with dietary diversity among anemic pregnant women. In this study, maternal age, education level, income, meal frequency, calcium, and iron adequacy levels were not significantly associated with dietary diversity (Table 5).

## 4. Discussion

This study found that among 152 anemic pregnant women, 42.8% were moderately anemic. This proportion was lower than that of the pregnant women in Indonesia [3], rural India [17], and West and Central Africa Region [18]. Nonetheless, it was significantly higher than that of anemic pregnant women in North Sumatera, Indonesia [19]. Maternal anemia may have a negative effect on birth outcomes such as stillbirth, neonatal death, and low birth weight. The risk increases with anemia severity [17].

This cross-sectional study discovered that family size, gestational age, and nutrient adequacy (energy, protein, vitamin A, vitamin C, and zinc) were associated with dietary diversity among anemic pregnant women. The anemic pregnant women with smaller family size and younger gestational age tended to have a more diverse diet. Larger family size was negatively associated with dietary diversity. A study by Gigatia et al. [20] in high-potential agricultural areas in Kenya found that the number of household members could affect dietary diversity through intra-household distribution and limited access to consumption of various kinds of food groups.

Pregnant women in their first trimester had a higher dietary diversity compared to those in the second and third trimester. Suffering nausea and vomiting, decreasing appetite, and feeling tired in early pregnancy were common complaints among anemic pregnant women. Therefore, pregnant women in trimester 1 consume food not causing nausea in small amount. Based on food recall, women in early pregnancy tend to consume small amount of traditional mixed dishes that contain more than 3 food groups like *rujak lontong*, *rujak buah*, and *es campur*. This dishes contain fruits and vegetables, grains, meat, poultry, and fish. Higher consumption of milk, meat product, and eggs was also found among women in their first trimester. Pregnant women in the second and third trimesters consume traditional mixed dishes in greater amount but low proportion of consumption of milk, meat product, and eggs. Pregnant women who consumed ≥4 food groups during pregnancy had a lower risk of anemia, low birth weight, and preterm delivery [12]. Based on severity, mildly anemic had a more diverse diet than moderately anemic pregnant women. Mildly anemic pregnant women consume more animal protein (eggs, milk, and dairy product), vegetables, and fruits than moderately anemic pregnant women. The result of the study implies that diverse food consumption needs to be taken into account by pregnant women particularly with moderate anemia.

Similar to our study, Ali et al. in Pakistan [21] also found that age, education level, and income were not significantly correlated with dietary diversity. However, it had a different result with Kiboi et al. in Kenya [13]. It might occur because of the differences in the samples used. The respondents in this study were anemic pregnant women, while other studies used a combined sample of anemic and nonanemic pregnant women.

The present study showed that nutrient adequacy (energy, protein, vitamin A, vitamin C, and zinc) was positively correlated with dietary diversity. High dietary diversity can lead to adequate nutrient intake. More than half of anemic pregnant women reached their minimum dietary diversity with a median of five food groups. The dietary diversity was lower than that of pregnant women in Kenya [13] but higher than that of pregnant women in Bangladesh [22]. The pregnant women had given attention to minimum dietary diversity but not the dietary quantity. The anemic pregnant women mostly consumed cereals, animal food (fish), and pulses (tempeh and tofu), dark-green leafy vegetables, and eggs. Dietary recall data revealed that pregnant women in Madura were used to consume mixed dishes such as *lontong campur*, *ketupat sayur*, *lontong mie*, *rujak lontong*, *rujak buah*, and *es campur*. These traditional mixed dishes contain more than two or three food groups. The Indonesian Dietary Guidelines strongly recommend the importance of consuming a variety of foods needed to fulfill the nutritional needs [23].

Pregnant women were advised to consume many kinds of food sources of carbohydrates, protein, vitamins, and minerals such as rice and rice corn (*nasi jagung*), fish, pulses (tempeh and tofu), vegetables, and fruits [24]. Low consumption of milk needs to be a concern for pregnant women. Milk is a good source of protein and minerals. A prospective cohort study by Olsen et al. [25] revealed that milk consumption was associated with higher birth weight. However, Melnik et al. [26] were concerned about the increasing birth weight that could be a risk factor for the development of civilization diseases. Therefore, they suggested defining save upper limits for milk consumption during pregnancy, particularly for women with high prepregnancy BMIs.

Adequate nutrition is needed by pregnant women. However, low dietary quantity was prevalent among anemic pregnant women in this study. A systematic review by Harika et al. in African countries also found inadequate intakes of micronutrients among pregnant women [27]. Most of nutrient adequacy levels were below the recommendation, except for iron. Anemic pregnant women mostly ate ≤3 times/day. Based on 2 × 24-h dietary recall, the small eating portion was commonly found among respondents. The majority of pregnant women only consumed food sources of carbohydrates with side dishes (e.g., tofu and tempeh) or with small slices of fish or chicken egg. Nutritional requirements during pregnancy increased due to physiological, metabolic, and anatomic changes. The additional requirements were used for the formation of new cells and tissues and also to fulfill the need for energy to support the activities of pregnant women and the fetus growth [28].

The iron adequacy level was the only micromineral fulfilled by pregnant women. The consumption of iron-folic acid (IFA) supplement increases the iron adequacy level. Food contributes only 15–20% to iron adequacy level. During pregnancy, the pregnant women in Indonesia receive 90 IFA tablets for free from health personnel to anticipate anemia. Multivitamin and multi-micronutrient supplementation can be one of the alternatives in solving the nutritional problem among pregnant women instead of only distributing IFA supplement. Based on the Cochrane database of systematic reviews, pregnant women who received multi-micronutrient supplementation had fewer low-birth-weight infants and small-for-gestational-age (SGA) infants than those receiving only iron supplementation, with or without folic acid. Therefore, the replacement of iron and folic acid with multi-micronutrient supplements for pregnant women in low- and middle-income countries where multi-micronutrient deficiencies are prevalent among women is important [29]. Pense et al. stated in their review that multi-micronutrient supplement reduced the risk of low birth weight and SGA but had no significant effect on preterm birth, miscarriage, stillbirth, and overall neonatal mortality [30]. The WHO has stated that multi-micronutrient supplementation is not recommended for pregnant women to improve maternal and perinatal outcomes. However, the use of the supplement in a population with a high prevalence of nutrient deficiencies may need to be considered because the benefits of the multi-micronutrient supplement for maternal health outweigh its disadvantages [31]. The Centers for Disease Control and Prevention recommend the multivitamin supplementation for pregnant women who do not consume an adequate diet [30].

### 4.1. Study Limitation

This descriptive study revealed no severe anemic pregnant women. Therefore, this study cannot describe the dietary quantity and diversity in all anemia categories. Dietary recall depends on participants' memory and elaboration of food or household size. Therefore, food recall was done by the trained nutritionist in nonconsecutive days using the food picture book. Probing with daily activity also done by the enumerators to help the participants remember the food they were consume. Analytical study that includes all anemia severity (mild-severe) among pregnant women with another dietary assessment method (food weighing) can be a prospective study in order to understand the correlation of dietary quantity and diversity.

## 5. Conclusion

Poor dietary quantity among mild and moderate anemic pregnant women was common in this study. Consumption of low dietary diversity was associated with energy, and macro- and micronutrients. Anemic pregnant women with moderate anemia, in their third trimester, and those with a large family are vulnerable to have a low dietary diversity and quantity. Attention needs to focus on increasing consumption of animal protein (eggs, milk, and dairy product), vegetables, and fruits with enough quantity, so the fulfillment of nutrient adequacy can be achieved.

## Figures and Tables

**Table 1:** Characteristics of anemic pregnant women.

Variables	Anemia severity		Total
	Mild	Moderate	
Age (years)			
≤18	2 (33.3)	4 (66.7)	6 (100)
19–29	61 (59.8)	41 (40.2)	102 (100)
30–49	24 (54.5)	20 (45.5)	44 (100)
Education level			
Basic (≤9 years)	32 (48.5)	34 (51.5)	66 (100)
Secondary (10–12 years)	34 (60.7)	22 (39.3)	56 (100)
Tertiary (>12 years)	21 (70.0)	9 (30.0)	30 (100)
Family size			
Small (≤4 people)	50 (58.8)	35 (41.2)	85 (100)
Medium (5–7 people)	30 (54.5)	25 (45.5)	55 (100)
Large (>7 people)	7 (58.3)	5 (41.7)	12 (100)
Income			
Quintile 1	24 (63.2)	14 (36.8)	38 (100)
Quintile 2	27 (71.1)	11 (28.9)	38 (100)
Quintile 3	19 (50.0)	19 (50.0)	38 (100)
Quintile 4	17 (44.7)	21 (55.3)	38 (100)
Pregnancy stages			
Trimester 1	8 (61.5)	5 (38.5)	13 (100)
Trimester 2	30 (54.5)	25 (45.5)	55 (100)
Trimester 3	49 (58.3)	35 (41.7)	84 (100)
Meal frequency			
≤2x/day	22 (47.8)	24 (52.2)	46 (100)
3x/day	55 (61.1)	35 (38.9)	90 (100)
≥4x/day	10 (62.5)	6 (37.5)	16 (100)

**Table 2:** Health complaints of anemic pregnant women.

Health complaints	Anemia severity		Total
	Mild	Moderate	
Back pain			
Yes	54 (54.0)	46 (46.0)	100 (100)
No	33 (63.5)	19 (36.5)	52 (100)
Feeling tired			
Yes	48 (52.2)	44 (47.8)	92 (100)
No	39 (65.0)	21 (35.0)	60 (100)
Upper respiratory tract infection			
Yes	36 (50.7)	35 (49.3)	71 (100)
No	51 (63.0)	30 (37.0)	81 (100)
Head ache			
Yes	38 (54.3)	32 (45.7)	70 (100)
No	49 (59.8)	33 (40.2)	82 (100)
Nausea/vomiting			
Yes	35 (56.5)	27 (43.5)	62 (100)
No	52 (57.8)	38 (42.2)	90 (100)
Decreasing appetite			
Yes	28 (53.8)	24 (46.2)	52 (100)
No	59 (59.0)	41 (41.0)	100 (100)
Constipation			
Yes	14 (56.0)	11 (44.0)	25 (100)
No	73 (57.5)	54 (42.5)	127 (100)
Swollen feet			
Yes	5 (50.0)	5 (50.0)	10 (100)
No	82 (57.7)	60 (42.3)	142 (100)

**Table 3:** Nutrition adequacy level of anemic pregnant women.

Nutrition adequacy level	Anemia severity		Total	*p* value
	Mild	Moderate		
Energy				
<70% RDA	70 (59.8)	47 (40.2)	117 (100)	0.636
70–79% RDA	6 (35.3)	11 (64.7)	17 (100)	
80–89% RDA	3 (42.9)	4 (57.1)	7 (100)	
90–110% RDA	5 (100)	0 (0)	5 (100)	
>110% RDA	3 (50.0)	3 (50.0)	6 (100)	
Protein				
<70% RDA	32 (55.2)	26 (44.8)	58 (100)	0.880
70–79% RDA	16 (64.0)	9 (36.0)	25 (100)	
80–89% RDA	8 (47.1)	9 (52.9)	17 (100)	
90–110% RDA	17 (56.7)	13 (43.3)	30 (100)	
>110% RDA	14 (63.6)	8 (36.4)	22 (100)	
Vitamin A				
<77% RDA	52 (55.9)	41 (44.1)	93 (100)	0.794
≥77% RDA	35 (59.3)	24 (40.7)	59 (100)	
Vitamin C				
<77% RDA	54 (54.0)	46 (46.0)	100 (100)	0.652
≥77% RDA	33 (63.5)	19 (36.5)	52 (100)	
Calcium				
<77% RDA	73 (57.0)	55 (43.0)	128 (100)	0.904
≥77% RDA	14 (58.3)	10 (41.7)	24 (100)	
Iron				
<77% RDA	12 (40.0)	18 (60.0)	30 (100)	0.148
≥77% RDA	75 (61.5)	47 (38.5)	122 (100)	
Zinc				
<77% RDA	79 (56.8)	60 (43.2)	139 (100)	0.955
≥77% RDA	8 (61.5)	5 (38.5)	13 (100)	

Mann–Whitney *U* Test (*α* < 0.05).

**Table 4:** Dietary diversity of anemic pregnant women.

Variables	Anemia severity		Total	*p* value
	Mild	Moderate		
Dietary diversity				
<5 food groups	32 (50)	32 (50)	64 (100)	**0.025**
≥5 food groups	55 (62.5)	33 (37.5)	88 (100)	
Staple foods (grains, roots, and tubers)				
Yes	87 (57.2)	65 (42.8)	152 (100)	1.000
No	0 (0)	0 (0)	0 (0)	
Animal food				
Meat, poultry, fish, and other seafoods				
Yes	69 (57.5)	51 (42.5)	120 (100)	0.899
No	18 (56.3)	14 (43.8)	32 (100)	
Eggs				
Yes	44 (60.3)	29 (39.7)	73 (100)	0.468
No	43 (54.4)	36 (45.6)	79 (100)	
Milk and dairy products				
Yes	13 (65)	7 (35)	20 (100)	0.453
No	74 (56.1)	58 (43.9)	132 (100)	
Plant food				
Pulses (beans, peas, and lentils)				
Yes	63 (57.8)	46 (42.2)	109 (100)	0.824
No	24 (55.8)	19 (44.2)	43 (100)	
Nuts and seeds				
Yes	32 (59.3)	22 (40.7)	54 (100)	0.709
No	55 (56.1)	43 (43.9)	98 (100)	
Vegetables and fruits				
Dark-green leafy vegetables				
Yes	49 (62.8)	29 (37.2)	78 (100)	0.154
No	38 (51.4)	36 (48.6)	74 (100)	
Vitamin A-rich fruits and vegetables				
Yes	26 (70.3)	11 (29.7)	37 (100)	0.066
No	61 (53)	54 (47)	115 (100)	
Other vegetables				
Yes	30 (63.8)	17 (36.2)	47 (100)	0.273
No	57 (54.3)	48 (45.7)	105 (100)	
Other fruits				
Yes	29 (61.7)	18 (38.3)	47 (100)	0.458
No	58 (55.2)	47 (44.8)	105 (100)	

Mann–Whitney *U* Test (*α* < 0.05).

**Table 5:** Association between characteristics and dietary quantity with dietary diversity of anemic pregnant women.

Variables	Median (IQR)	*r*	*p*
Age (years)	25 (8)	0.055	0.497
Education level (years)	12 (3)	0.117	0.152
Family size (people)	4 (2)	**−0.160**	**0.048**
Income (IDR/cap/month)	451,785 (350,397)	0.125	0.125
Meal frequency (x/day)	3 (1)	0.077	0.343
Gestational age (months)	26 (10)	**−0.164**	**0.044**
Energy adequacy level (%)	57.6 (23.9)	**0.177**	**0.029**
Protein adequacy level (%)	76.6 (42.2)	**0.238**	**0.003**
Vitamin A adequacy level (%)	51.4 (115.5)	**0.263**	**0.001**
Vitamin C adequacy level (%)	50.1 (79.5)	**0.233**	**0.004**
Calcium adequacy level (%)	28.6 (47.4)	0.143	0.078
Iron adequacy level (%)	181.8 (89.9)	0.103	0.206
Zinc adequacy level (%)	34.9 (27.7)	**0.197**	**0.015**

Spearman's rank correlation (*α* < 0.05).

## Data Availability

The data used to support this article are available at RIN Dataverse (https://data.lipi.go.id).
